# Implementing telehealth to support medical practice in rural/remote regions: what are the conditions for success?

**DOI:** 10.1186/1748-5908-1-18

**Published:** 2006-08-24

**Authors:** Marie-Pierre Gagnon, Julie Duplantie, Jean-Paul Fortin, Réjean Landry

**Affiliations:** 1 Evaluative Research Unit, Quebec University Hospital Centre, Quebec, Canada; 2 Department of Family Medicine, Laval University, Quebec, Canada; 3 Department of Social and Preventive Medicine, Laval University, Quebec, Canada; 4 Department of Management, Laval University, Quebec, Canada

## Abstract

**Background:**

Telehealth, as other information and communication technologies (ICTs) introduced to support the delivery of health care services, is considered as a means to answer many of the imperatives currently challenging health care systems. In Canada, many telehealth projects are taking place, mostly targeting rural, remote or isolated populations. So far, various telehealth applications have been implemented and have shown promising outcomes. However, telehealth utilisation remains limited in many settings, despite increased availability of technology and telecommunication infrastructure.

**Methods:**

A qualitative field study was conducted in four remote regions of Quebec (Canada) to explore perceptions of physicians and managers regarding the impact of telehealth on clinical practice and the organisation of health care services, as well as the conditions for improving telehealth implementation. A total of 54 respondents were interviewed either individually or in small groups. Content analysis of interviews was performed and identified several effects of telehealth on remote medical practice as well as key conditions to ensure the success of telehealth implementation.

**Results:**

According to physicians and managers, telehealth benefits include better access to specialised services in remote regions, improved continuity of care, and increased availability of information. Telehealth also improves physicians' practice by facilitating continuing medical education, contacts with peers, and access to a second opinion. At the hospital and health region levels, telehealth has the potential to support the development of regional reference centres, favour retention of local expertise, and save costs. Conditions for successful implementation of telehealth networks include the participation of clinicians in decision-making, the availability of dedicated human and material resources, and a planned diffusion strategy. Interviews with physicians and managers also highlighted the importance of considering telehealth within the broader organisation of health care services in remote and rural regions.

**Conclusion:**

This study identified core elements that should be considered when implementing telehealth applications with the purpose of supporting medical practice in rural and remote regions. Decision-makers need to be aware of the specific conditions that could influence telehealth integration into clinical practices and health care organisations. Thus, strategies addressing the identified conditions for telehealth success would facilitate the optimal implementation of this technology.

## Background

Telehealth is considered a major innovation at the technological, social, and cultural levels[1]. This technology has the potential to increase access to, and quality of, health care services and to lower health system expenditures[2,3]. Thus, introducing telehealth as a tool to support the delivery of health care services implies numerous changes for providers, organisations, and the health system as a whole that must be accounted for during the implementation process[4].

According to systematic reviews[2,3,5,6], evidence of telehealth benefits has been reported for various applications such as teleradiology, telepsychiatry, transmission of echocardiograms, teledermatology, and telehomecare. Results from a majority of the reviewed studies support telehealth over other traditional modes of health services delivery. Other studies have reported telehealth benefits with respect to continuity of patient care and coordination of clinical activities between various health care organisations and levels of care [7-10].

For rural, remote or isolated regions, telehealth is considered as a tool that could exert a positive impact on several dimensions of health care services delivery. For instance, telehealth can support the delivery of specialised services in a timely fashion for remote populations, facilitate access to education for clinicians, and save travel costs for patients and professionals. Moreover, as telehealth technologies become more integrated into the health care system, they could increasingly contribute to the reorganisation of medical workforce supply and exert a profound influence on physician practice, especially in remote areas[11].

Successful telehealth implementation represents the first step towards the normalisation of this technology as a means of health care delivery. According to May et al[12], normalisation is "*the move toward the routinized embedding of telemedicine in everyday clinical practice*" (p.596). Nevertheless, telehealth implementation still faces major barriers, mostly related to structural, organisational, and professional imperatives[4,13]. Specifically, structural barriers relate to licensure, reimbursement, policies governing telecommunication and information technologies development, and interjurisdictional collaborations[14,15]. Issues regarding health care organisations are also of paramount importance to ensure telehealth adoption. The introduction of a new technology challenges existing structural and operational features and a mutual adjustment is often required between the technology and the organisation[16,17]. Additionally, physicians represent one of the main groups of telehealth users and the introduction of this technology into their practice is affected by particular characteristics of the medical profession[18]. Furthermore, telehealth adoption by an individual is considered as a complex behaviour determined by a large set of psychosocial factors[19,20].

Knowledge is still limited with respect to the specific impacts of telehealth on the practice of health care professionals in rural and remote regions. A recent survey found no direct effects of telehealth on recruitment and retention of physicians in a rural area of Canada[21]. Nonetheless, this study indicated that rural physicians who used telehealth had a more positive perception of the value of this technology for their community. A study conducted among medical residents in Quebec found a significant correlation between residents' positive evaluation of telehealth and their intention to practice in a remote region[22]. However, given the complex and multiple influences on physicians' choice of practice location, it remains difficult to assess the specific contribution of telehealth on medical practice in rural and remote regions.

Telehealth benefits seem obvious for large territories with relatively dispersed population such as rural and remote regions of the Province of Quebec (Canada). Several telehealth projects have been implemented in Quebec over the last decade. Eastern Quebec was one of the first region to participate in telehealth demonstration projects, with the *Réseau de Télémédecine de l'Est du Québec *(Eastern Quebec Telemedicine Network: teleradiology and telecardiology), Projet de Démonstration de la Télésanté aux Iles-de-la-Madeleine (Magdalene Islands Telehealth Demonstration Project: multiple applications) and the *Réseau Québécois de Télésanté de l'Enfant *(Quebec Child Telehealth Network: paediatrics telecardiology). Evaluations of these projects have generally reported a positive impact on several factors related to the quality of clinical practice and the continuity of patient care in rural, remote or isolated regions [23-25]. However, these three projects failed to normalise in their initial form.

The reasons that may explain why most telehealth applications have failed to normalise in Quebec may be similar to those reported in other settings[12]. First, most were small scale demonstration or pilot projects that aimed to establish feasibility, safety, effectiveness, and conditions of use for an eventual diffusion of telehealth. In these projects, several telehealth applications were tested that formed a complex system of interactions between technologies, functionalities, information workflow, and users[25].

Second, telehealth projects are complex, innovative, constantly evolving, and many of their effects cannot necessarily be anticipated, which is then a challenge for the evaluation of their various impacts on the health care system[12,26]. Moreover, high quality evidence on 'what works' from well-designed experimentations such as randomised controlled trials (RCT) is not always sufficient to set up policies. Knowledge on the specific conditions that lead to telehealth normalisation in a given context ('how it works') is also essential[13]. Finally, evidence about global impacts of telehealth on health care professionals' work as well as on the recruitment and retention of medical workforce in remote regions is lacking.

Nevertheless, the Quebec Ministry of Health has identified telehealth as one of the means to counterbalance the uneven distribution of the medical workforce in the Province and an essential component of the reorganisation of health services[27]. Many telehealth projects are currently ongoing in Quebec, but telehealth is still not integrated as a routine service in the health care system.

In order to better understand the conditions promoting or limiting telehealth integration, this study explored telehealth's effects on several dimensions related to the practice of health care professionals in rural and remote regions of Quebec. Decision-makers were involved in the different phases of the research project in order to facilitate knowledge sharing and utilisation. A qualitative field study was conducted in four regions of Eastern Quebec to identify the perceptions of physicians and managers regarding telehealth benefits and limitations as well as the key conditions for successful telehealth implementation.

## Methods

### Selection of respondents

A purposive sampling technique was used to identify potential respondents[28]. In order to ensure both diversity of opinions and relevance to the topic of interest, selection criteria included: localisation, profession (clinician or manager), medical speciality, and telehealth experience (extensive or limited). The four regions of Eastern Quebec were chosen because of their involvement in previous telehealth experimentations, especially in teleradiology and telecardiology. Thus, professionals and managers of these regions were more knowledgeable about telehealth and its effects. These regions also combined various practice settings: urban hospitals, semi-urban hospitals, and rural and remote health centres.

Initial subjects were identified through personal contacts from members of the research team, lists of telehealth conference participants, and documentation on telehealth projects. Other potential respondents were identified through the snowball method which solicits referrals from initial participants to generate additional subjects[29]. Some medical specialists such as radiologists, cardiologist and paediatricians had experience in telehealth activities. Other respondents only used it for tele-education or meetings, whereas some respondents never used telehealth at all. Principles of data saturation and information redundancy were applied to determine sample size, i.e. the recruitment of participants ended when additional interviews did not bring new information or opinion[30].

### Development of the survey instrument

Interview schemas were elaborated from the literature and previous research done by the team[16,19,25]. A different schema was prepared for clinicians and managers. These schemas were pre-tested with four collaborators of the research team who had medical and/or management backgrounds.

The interview schema for physicians was divided into three parts. The first part comprised questions about actual practice, motivations for practicing in the remote region, motivations for staying in the region, as well as potential factors that could make one leave the region. The second part dealt with the quality of life at work and the effects of telehealth on clinical practice. If respondents did not have access to telehealth, perceptions concerning its possible applications to their practice were gathered. The last part of the interview covered perceptions about the benefits and limitations of telehealth use in one's practice as well as the conditions that would facilitate telehealth integration into clinical work.

Managers from hospitals and health regions were also interviewed about the nature of their work and the strategies they were using to attract and keep medical workforce in the region. Managers were also asked questions dealing with the effects of telehealth on clinical practice and organisation of care. Finally, questions addressed their opinion about telehealth benefits and limitations, as well as their perceptions about requirements to ensure telehealth integration.

### Data collection procedures

A research professional contacted potential respondents by telephone to present the study and to solicit their participation in an interview. For logistic reasons, interview scheduling was limited and potential respondents had to be available on the day planned for the visit to their centre. Those who were interested and available received a copy of the schema by electronic mail during the week prior to interview.

Written consent was obtained from all respondents prior to interview. Interviews lasted from 20 minutes to one hour. For logistic purposes, some interviews were conducted in small groups of two to five persons. All interviews were tape-recorded with the consent of respondents and a verbatim transcript was made. Two researchers trained in social and health sciences conducted the interviews and gathered observation notes. This material was used together with interviews content for analyses.

### Data analysis

A qualitative iterative strategy was adopted for data analysis, based upon the method proposed by Huberman and Miles[31]. In a first step, all interview transcripts and field notes were read to extract general impressions and preliminary classification categories. Seven broad categories were created: 1) recruitment factors; 2) retention factors; 3) quality of life at work; 4) telehealth benefits; 5) telehealth limitations; 6) conditions for telehealth integration; and 7) potential impact of telehealth on recruitment and retention. In a second step, two researchers classified interview content into matrixes corresponding to these categories. Using an iterative approach, emerging patterns and themes were identified within each category and discussed between the researchers. After a consensus on coding themes, content was independently coded by the two researchers. Analyses were then compared and adjusted after a consensus discussion with the research team.

To assess divergences and convergences between physicians' and managers' views, as well as to gain an overview of the qualitative material, the weight of each theme was assessed by the frequency of its being mentioned. Only themes mentioned by three respondents or more were considered in this analysis. Aspects related to physician recruitment and retention are beyond the scope of this article; only findings related to perceptions about telehealth benefits and limitations, as well as conditions to facilitate telehealth integration into practices are presented and discussed below.

### Ethical approval

The study received approval from the ethics committee of the Quebec University Medical Centre.

## Results

A total of 40 physicians and 14 managers were interviewed.

### Perceived telehealth benefits

For a majority of physicians and managers, telehealth was perceived as a powerful tool to improve healthcare services for populations living in remote areas. According to respondents, telehealth has the potential to facilitate access to, and availability of, services that would be difficult to obtain otherwise. As shown in Table 1, many respondents agreed that telehealth implementation has brought specialised services to patients close to their home and that many transfers were avoided, saving significant travel costs for patients and their family. Moreover, respondents have reported that telehealth could be helpful to transmit information before transferring a patient to an urban centre, thus facilitating case management. Telehealth is also viewed as an efficient means to perform follow-up visits in order to improve continuity of care. In some cases, telehealth can also allow a first evaluation of a remote patient by a visiting specialist: "*There's a paediatric specialist who comes only once a year and he asked to use telemedicine for his first evaluation of a new patient so that his visit would be improved. That way he can operate kids who otherwise would have to wait much longer*." (Hospital manager, region 10).

**Table 1:** Perceived telehealth benefits and limitations

**Dimension**	**Perceived Benefits **(*Frequency*)*	**Perceived Limitations **(*Frequency*)*
Clinical/Patient care	Access to specialised services (*5 md, 9 hm*) Potential to save costs for patients (*3 md, 4 hm*) Facilitates management of transfers (*4 md*) Allows distant follow-up that improves continuity of care (*3 md*) Improves information circulation (*3 md*)	Telehealth will never replace on site physician (*6 md, 1 hm*)
Professional	Access to a second opinion (*10 md, 2 hm*) Facilitates communication with peers (*7 md, 3 hm*) Diminishes the feeling of isolation (*3 md, 2 hm*)	Anticipated changes in the definition of tasks and responsibilities (*2 md, 2 hm*)
Educational	Knowledge development and update (*7 md, 2 hm*) Increases access to CME (*4 md, 4 hm*) Multi-disciplinary/multi-centered exchanges (*3 md*)	Teleeducation cannot substitute for all CME activities (*2 md, 1 hm*)
Organisational/Systemic	Supports the hospital as a regional reference centre (*6 md, 5 hm*) Ensures availability of services (*4 md, 3 hm*) Saves time and money for meetings (*4 md, 3 hm*) Potential to save costs for health system (*3 md, 4 hm*) Better organisation of on-call duties (*4 md*)	Fear of replacing regional specialists (*3 md, 2 hm*) Heavy logistics needed in the two sites (*2 md, 2 hm*) Lack of commitment from the organisation (*2 md, 1 hm*)

* Number of physicians (*md*) and hospital managers (*hm*) who mentioned the item.

At the professional level, telehealth was perceived as an excellent means of communication for remote physicians by providing them with easy access to a second opinion and contacts with their peers. Feelings of isolation are common among remote physicians[32]. Thus, for a physician working in a remote region, telehealth could act as a way to keep in touch with peers and colleagues from other regions. Telehealth was also considered as an efficient way to provide education and to facilitate exchanges between professionals from various sites and specialties. "*It's more difficult to work with a remote specialist if you don't know him or her. That's why we've asked for an affiliation with another centre, like the one we have with *[hospital's name] *through continuing medical education. We've asked to get 'live' access to videoconferences from all over the province*." (Medical specialist, region 09). Another telehealth benefit identified was the possibility to organise on-call duties on a regional basis for specialties such as radiology. Thus, instead of sharing the responsibility for on-call duties between radiologists of a single hospital, telehealth could allow a greater number of specialists from different centres to cover the whole region. "*Six months ago, we began regional on-call duty covering three hospitals of the region. We are four radiologists who share the responsibility each week*." (Medical specialist, region 01).

Hospitals and health care centres located in remote regions can also benefit from telehealth since it offers a support to ensure the complete coverage of population needs in terms of health care services. "*With telehealth we can have access to ultra specialised services without transferring the patient. The idea is not transferring patients if we can offer the service here. It doesn't make sense to transfer a patient only for a diagnosis when it can be done remotely*." (Hospital manager, region 09). Hence, telehealth could allow for the development of regional reference centres that would provide a wide range of services to remote populations. However, this situation could also create competition between hospitals of a same region for obtaining the status of a referral centre. Moreover, telehealth is believed to produce significant savings for remote hospitals and for the health care system. For instance, teleconference can be used to attend administrative meetings, leading to substantial savings on travel costs. However, the redistribution of savings between organisations and levels of care is an important and complex issue.

### Perceived telehealth limitations

As shown in Table 1, only few limitations were reported with respect to the use of telehealth in remote regions. For instance, physicians were concerned about the fact that telehealth could replace onsite human resources. Respondents also commented that some specialists would prefer to stay in university centres and to provide services via telehealth rather than moving to a remote region: "*Telemedicine could make people want to stay where they are, in university centres, but it won't replace a radiologist in the region, who can be in contact and play a different role as consultant with other physicians*." (Hospital manager, region 02). A similar fear was present that tele-education through videoconference would replace all continuing medical education (CME) activities outside the region. For remote physicians, participation in scientific activities in urban centres also represents an occasion to socialise with their colleagues, which could never be replaced by teleconferences.

### Perceived conditions for telehealth success

Physicians and managers were also asked to discuss the conditions that could help telehealth integration into their practice. Responses were classified into six dimensions representing the levels at which efforts would be needed to facilitate telehealth integration into practice. These findings are presented in Table 2. At the individual level, respondents agreed that telehealth should be easy to use and compatible with daily practice. As one physician said: "*The system must adapt to my practice and not vice-versa*." (Medical specialist, region 01).

**Table 2:** Conditions for telehealth implementation

**Dimension**	**Condition **(*Frequency*)*
Individual	Perceived ease of use (*4 md, 3 hm*) Technology integrated to the daily practice (*3 md, 2 hm*) Healthcare professionals' motivation (*2 md, 1 hm*)
Professional	System based on the needs of health care professionals (*4 md, 3 hm*) Adequate remuneration for professionals in both sites (*6 md, 1 hm*) Defining clear rules for professional liability (*3 md, 2 hm*) Participation of physicians in telehealth decision-making (*3md*)
Organisational	Availability of resources dedicated to telehealth (specialised nurses, technicians, etc) (*5 md, 4 hm*) Specific schedules for telehealth consultations (*3 md, 6 hm*) Referrals based upon existing collaboration networks (*3 md, 3 hm*) Availability of up-to-date equipment (*2 md, 3 hm*)
Socio-political/Systemic	Massive investments in technologies and infrastructures (*1 md, 4 hm*) Regional agreements and local development plans for health care services delivery based upon a combination of local expertise, outreach services, and access to specialists with telehealth (*1 md, 4 hm*)
Technological	Reliable, mobile, ergonomic, and user-friendly systems (*4 md, 5 hm*) Image quality to allow diagnosis (*4 md, 1 hm*)
Ethical/Legal	Ensuring data confidentiality (*2 md, 2 hm*)

* Number of physicians (*md*) and hospital managers (*hm*) who mentioned the condition.

Motivation of healthcare providers was also deemed important to facilitate telehealth integration. A successful telehealth network should be based upon clinicians' needs and promote their participation in decision-making. "*We must be involved in the whole implementation process*." (General practitioner, region 10). This reflects a bottom-up implementation strategy where end-users are first consulted to identify their needs and expectations, followed by an iterative approach where they are involved in decisions at different stages of the project development.

Healthcare organisations also have an important role in supporting telehealth integration. Human, material, and logistical resources need to be provided to ensure the functioning of telehealth services. Moreover, at the socio-political level, it is important to secure financing for equipment maintenance and upgrade. At the technological level, the various components of telehealth systems must correspond to users' expectations in terms of reliability, ergonomics, mobility, and user-friendliness. Finally, telehealth networks should assure the required level of security in order to protect data confidentiality and patient privacy.

## Discussion

This study aimed to explore the potential of telehealth to support medical practice in rural and remote regions, as well as the conditions to ensure successful implementation of this technology into health care organisations. Telehealth shows several potential benefits for rural and remote populations and could definitely improve patient care as a result of increased accessibility to specialised services, better continuity of care, and avoided transfers. These findings confirm those reported in other telehealth projects[3,6,25]. However, despite growing evidence of its benefits, telehealth is not yet integrated as part of the everyday medical practice in the Quebec health care system. Using the framework of telehealthcare normalisation proposed by May et al[12], there are many conditions that need to be addressed in order to facilitate telehealth integration into routine care.

First, remote and rural physicians are among the principal telehealth users. It is thus important to emphasise telehealth's benefits on the work of health care professionals. Telehealth has the potential to improve work satisfaction by providing easier access to continuing education and facilitating contacts with colleagues. Access to CME has been associated with higher satisfaction at work and better quality of care[32] and could be a factor of physician retention in rural and remote areas[33]. Such findings can support decision-making with respect to the diffusion of telehealth services in remote regions. However, there is also a potential threat that telehealth would encourage specialists to stay in urban centres while using telehealth to provide coverage to remote regions. This has been pointed out as a possible consequence of the diffusion of teleradiology services[34].

Second, telehealth is also seen as a means to support the organisation of health care delivery on a regional basis, allowing greater access to specialised resources and better distribution of on-call duties between physicians from a whole region. Therefore, an indirect impact of telehealth is an increased autonomy for rural and remote regions. This could generate some tension between regions and levels of care since specialised services could be directly accessed via telehealth instead of following the usual referral process to the regional hospital. It is thus important to respect usual referral patterns when implementing telehealth.

Third, the involvement of key stakeholders representing professional groups, health care organisations, and health regions in the planning and development of telehealth networks appear essential for achieving the normalisation of services. These findings are consistent with several studies on telehealth adoption [18-20]. Also, these findings support the importance of respecting existing collaborative networks between professionals in the referral processes since trust is an important element for telehealth success[35,36].

Finally, the concept of telehealth readiness has been proposed to describe the degree to which communities, organisations, and professionals are prepared to participate and succeed in telehealth[37]. Ideally, readiness should be assessed prior to the implementation of a telehealth project to reduce the risk of its failure after introduction[38]. Monitoring telehealth readiness can also indicate where specific efforts should be invested in order to facilitate the transition of telehealth from experimental to routine service. The factors identified in the present study could provide a basis to assess telehealth readiness in remote and rural regions of Quebec and other similar jurisdictions.

### Strengths and limitations

A set of criteria was applied to ensure the quality of the study process and results based upon recommendations for qualitative research [39-42]. First, some of the authors had certain preconceptions as researchers in the field of telehealth. However, a researcher with no such background was involved in the study. Furthermore, a steering committee of key stakeholders representing the Ministry of Health, medical associations, academia, and telehealth projects participated in the whole research process. These combined factors are likely to have improved the reflexivity and transparency of the study[40,41].

Second, study participants were selected to represent various points of view. Fifty-four physicians and managers, with extensive or limited telehealth experience, from four health regions participated in interviews and provided a broad range of opinions. Moreover, the results showed many similarities with those from other studies on telehealth implementation in Canada[36,37] and elsewhere[4,12]. The present findings are thus likely to apply to a variety of telehealth applications and to be transferable to similar settings[40].

Third, data classification and coding was conducted independently by two researchers and interpretations were compared for competing conclusions, thus improving the interpretative validity of the findings[42]. However, we did not use interviewee checking to ensure that our interpretation of the data were in line with what the participants had expressed[41]. Instead, a panel of experts from the project's steering committee was consulted and provided useful insight about the findings, thus ensuring the credibility and trustworthiness of the study[39].

Finally, emerging themes from interviews have been used as the basis for developing the questionnaire for a quantitative survey of the effect of telehealth on medical workforce recruitment and retention. Another contribution of this qualitative study was thus the generation of hypotheses to further assess the impact of telehealth on medical practice in rural and remote regions.

Telehealth research represents an emerging field; therefore theoretical and methodological developments are still needed in order to provide a common understanding of the implications of this technology at various levels of the health system. Different types of evidence are needed in order to influence decisions and policies regarding the diffusion of telehealth in the health care system. High quality evidence on telehealth effectiveness to support the delivery of health services in rural and remote regions is still needed. However, it is also important to understand the context in which telehealth is implemented in order to facilitate an optimal integration of this technology in practices and organisations.

## Conclusion

The success of telehealth implementation and its integration into routine health services depend upon several levers. First, taking into account physicians' needs and expectations is essential to the development of telehealth networks; their participation into decision-making is thus central. Second, health care organisations are required to allocate human and material resources in order to support telehealth activities. Generally, telehealth benefits are only visible over a long period of time while its development requires important investments on a short term. Successful telehealth implementation requires a progressive diffusion strategy, starting with applications that have proven benefits.

Overall, this study provides a comprehensive overview of key conditions that are essential for the implementation of telehealth applications in order to meet the expected benefits on patient care, professional practice, and organisation of services in rural and remote regions.

## Competing interests

The author(s) declare they have no competing interests.

## Authors' contributions

MPG and JD prepared interview guides, conducted interviews, analysed interview transcripts and coded data. MPG proceeded to the literature review and wrote a first draft of the manuscript. JPF and RL were co-Principal Investigators on the project and gave feedback on research instruments and data analyses. All four authors revised and approved the last version of the manuscript.
